# The crystal structure of the β subunit of luteinizing hormone and a model for the intact hormone

**DOI:** 10.1016/j.crstbi.2019.07.001

**Published:** 2019-08-12

**Authors:** Steven B. Larson, Alexander McPherson

**Affiliations:** Dept. Molecular Biology and Biochemistry, University of California, Irvine, Irvine, CA, USA

**Keywords:** Gonadotropic hormone, Pituitary hormone, Reproduction, Crystallography, Glycosylation, Proteolysis

## Abstract

The β subunit of bovine luteinizing hormone (LH) was crystallized and its structure solved to 3.15 ​Å resolution by molecular replacement using human chorionic gonadotropin (hCG) β subunit as search model. The asymmetric unit contains two copies of the β subunit that are related by a non-crystallographic symmetry (NCS) two-fold axis, both copies of which contain proteolytic cleavages after amino acid 100. It is noteworthy that the oligosaccharide moieties covalently attached at asparagine 13 were particularly pronounced in the electron density, allowing seven sugar residues to be defined. The α subunit of LH, which is common to all glycosylated gonadotropin hormones, was placed by superposition of hCG on the LH beta subunits, thereby yielding a model for the intact hormone.

## Highlights

• The X-ray crystallographic structure of the beta subunit of bovine luteinizing hormone (LH) has been determined at 3.15 A resolution
• A model for the entire LH has been proposed based on the beta subunit structure reported here and the known structure if the common alpha subunit
• A large amount (7 sugar residues on one molecule in the asymmetric unit, 5 on the other) of the oligosaccharide attached at asn 13 was visible in electron density maps
• Each of the two molecules in the asymmetric unit, which contains an NCS twofold axis, is associated with two molecules of beta octylglucoside
• The beta subunit has a proteolytic cleavage between amino acids 100 and 101, which releases the “seatbelt” peptide, though that peptide remains associated through an S–S bridge to the core.

## Graphical abstract

A model of luteinizing hormone based on the known α subunit and the β subunit determined here by X-ray crystallography.

**Figure:**
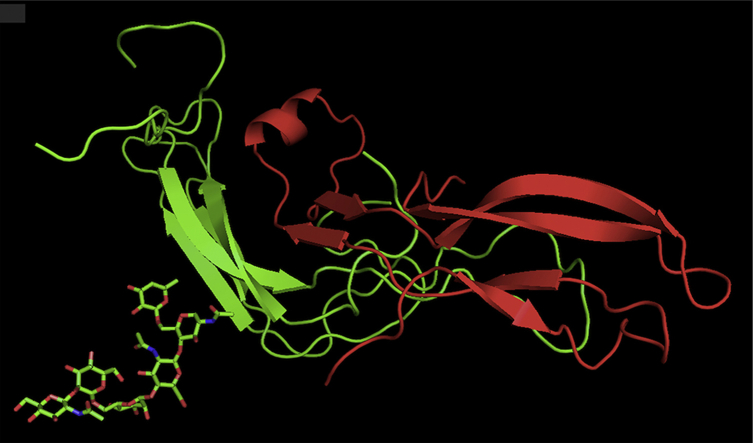

## 1 Introduction

There are in mammals four glycosylated gonadotropic hormones (GPH) that are produced in the anterior pituitary gland, three of which are involved in reproduction. They are chorionic gonadotropin (CG), which is produced in the developing conceptus, follicle stimulating hormone (FSH), and luteinizing hormone (LH). The fourth hormone is thyroid stimulating hormone (TSH) (Jiang et al., 2014, Talmadge et al., 1984). The crystal structure of hCG (1HCG) was solved in 1994 (Wu et al., 1994, Lapthorn et al., 1994, Tegoni et al., 1999) that of FSH (1FL7) in 2004 (Fox et al., 2001) in complex with the hormone binding portion of its receptor FSHR (Fan and Hendrickson, 2005, Fan and Hendrickson, 2007, Jiang et al., 2014). The structures of LH and TSH have not been determined. Although the four polypeptide hormones share only about 35% sequence identity, crystal structures, along with other evidence, confirm that the overall structures of the four hormones, which apparently evolved from a common gene (Talmadge et al., 1984), are highly similar (Jiang et al., 2014).

GPH actively exist as heterodimers consisting of a common α subunit and a unique β subunit that confers receptor specificity. The structure of the common α subunit is known from previous crystallographic analyses. Both the α and β subunits of GPH belong to the cysteine– knot growth factor family of polypeptides. All β subunits contain six disulfide bridges (see supplemental Figure 1) that lock beta strands into a central, non-hydrophobic core with associated flexible exterior loops (McDonald and Hendrickson, 1993, McDonald et al., 1991). The hormones have an unusually high surface to volume ratio with minimal hydrophobic core (Jiang et al., 2014) and they are extensively glycosylated, each in a different way (Baenziger and Green, 1988). Following removal of a 20 amino acid signal peptide, the lengths of the β subunits of the GPH are hCG – 145 amino acids, FSH – 109 amino acids, LH – 121 amino acids, and TSH – 112 amino acids. The α subunit is, in all hormones, 92 amino acids in length and contains five disulfide bonds (UniProt Data Bank].

The β subunit of LH that we crystallized from PEG in the presence of β octyl glucoside and trypsin was a part of the protein preparation that was originally sequenced by Prof. John Pierce in his pioneering work at UCLA (Parsons et al., 1983, Pierce and Parsons, 1981, Pierce et al., 1976, Reeve and Pierce, 1981, Reeve et al., 1975). X-ray diffraction data were collected at SLAC in 1999, but the solution to the crystal structure eluded us until recently. Using algorithms and programs developed in the last twenty years, we were able to solve the structure by molecular replacement, with hCG (1HCN) (Wu et al., 1994) as a search model, and refine the structure using maximum likelihood (Murshudov et al., 2011, Murshudov et al., 1997, Read, 2001). The resolution of the data, which went to the limit of the diffraction pattern (corresponding to an overall thermal factor of about 125 ​Å^2^), is relatively low in comparison with current structure determinations. We attribute this to the unusual flexibility of the extended loops, and their partial disorder. It should also be noted that in addition to 10% of the residues being cysteines engaged in disulfide bonds, another 20% of the residues are prolines, occasionally as runs of multiple prolines in short stretches.

## 2 Materials and methods

LH β subunit was given to us in 1994 upon the retirement of Prof. John Pierce from UCLA who did most of the original work on this hormone, including its sequencing (Parsons et al., 1983; Pierce and Parsons, 1981; Pierce et al., 1976; Reeve and Pierce, 1981; Reeve et al., 1975). It was a lyophilized white powder that was maintained at −20 ​°C. After many discouragements, small, plate-like crystals were obtained in 1998. A 30 ​mg/ml solution of protein in 0.10 ​M sodium citrate at pH 5.8, and 1% β octyl glucoside (BOG) was incubated at 37 ​°C for 24 ​h with 0.1 ​mg/ml bovine trypsin (Calbiochem, San Diego, CA). The protein was then crystallized at 4 ​°C, over a period of several weeks, by sitting drop vapor diffusion (McPherson, 1999; McPherson, et al., 1986) against 0.6 ​ml reservoirs in Cryschem plates (Hampton Research, Aliso Viejo, CA) consisting of 20% w/v PEG 3350 and 1% BOG, buffered with 0.10 ​M sodium citrate at pH 5.8. The droplets were 6 ul volume composed of equal volumes of the protein solution, still containing trypsin and BOG, with reservoir solution.

Data were collected on beam line 7.1 ​at SLAC with a MAR CCD detector, processed, and scaled using the program d*Trek (Pflugrath, 1999). The crystals were of space group F222, untwined according to the L test (Padilla and Yeates, 2003), with cell dimensions a ​= ​80.02 ​Å, b ​= ​80.02 ​Å, c ​= ​206.13 ​Å, and diffracted to about 3.0 ​Å. The crystals had pseudo space group I4_1_22 with a ​= ​b ​= ​56.57 ​Å, c ​= ​206.13 ​Å. The volume to mass ratio for the crystals (Matthews, 1968) is 2.79 ​Å^3^/Da, implying a solvent content of 56%. Statistics for data collection and scaling are presented in Table 1.

**Table 1:** Crystal and data collection statistics.

Space group	F222
Cell dimensions in Å	a ​= ​80.02, b ​= ​80.02, c ​= ​206.13
Vm/% solvent	2.79 ​Å^3^/Da, 56%
Mol. asym. unit	2
Beamline/detector	SLAC 7.1, MAR CCD
Resolution of diffraction	60.0–3.0 ​Å
R_merge_	0.138 (0.56)
R_meas_	0.140 (0.57)
I/sigma	9.1 (3.3)
CC1/2 (0.30)	3.1 ​Å
Number of observations	60,480
Number unique reflections	6465
completeness	98.43 (100.0)
multiplicity	9.35 (9.59)

Using as search model the β subunit of human chorionic gonadotropin extracted from PDB entry 1HCN (Wu et al., 1994) from the Protein Data Bank (Bernstein et al., 1977), and the program PHASER (McCoy et al., 2005, McCoy et al., 2007) two copies of the LH β subunit, related by an NCS two-fold axis, were found as the asymmetric unit of the crystals. The proteins were rebuilt in accordance with the amino acid sequence of LH β [Uniprot P04651-LSHB_BOVIN]**,** and restrained refinement with TLS restraints added was carried out using the program REFMAC5 (Murshudov et al., 1997) of the CCP4 program system (CCP4, 1994). NCS restraints were maintained throughout. Final statistics are shown in Table 2. Model building, calculation of Fourier and difference Fourier maps, superposition of models, and model validation were done with the program COOT (Emsley and Cowtan, 2004). Figures were made using Pymol (DeLano, 2002).

**Table 2:** Refinement and geometric statistics.

Resolution	30.0–3.15 ​Å
Unique reflections^a^	5771
Free R set	5.0%
Working reflections	5482
R_working_	0.205 (0.231)
Rfree	0.274 (0.322)
r.m.s.d bond lengths	0.009 ​Å
r.m.s.d bond angles	1.8°
r.m.s.d chiral vol.	0.084 ​Å^3^
Ramachandran outliers	10, 5/molecule
Rotamer outliers	none
Water molecules	28
ligands	β octylglucoside (2)

a Data was cutoff at 3.15 ​Å resolution.

## 3 Results and discussion

The structure of the asymmetric unit of two β subunits of LH is shown in Fig. 1 where the presence of NCS twofold symmetry is evident. Also present were the oligosaccharides covalently attached at asparagine 13 and two molecules of β octyl glucoside, a non-ionic detergent. Though not uncommon (Ward et al., 1986), and consistent with the inclusion of trypsin in crystallization, neither of the two protein molecules are fully intact, though almost all of their amino acids are present in the structure. Both molecules contain a continuous stretch from the amino terminus, serine 1, to cysteine 100, which is linked by a disulfide bond to cysteine 93. There is then a break in the polypeptide chain between cysteine 100 and glycine 101. The carboxy terminal fragment is not, however, lost because it contains cysteine 110, which remains linked to cysteine 26 by a disulfide bridge. Thus, the strand gly101 through ile118 (amino acids 119–121 are not seen) is present but clearly does not represent its disposition in the intact β subunit. In hCG and FSH the portion of the strand from gly101 to cys110 constitutes what is known as the “seatbelt polypeptide” that wraps around and locks the α subunit to the β subunit (Xing et al., 2004, Jiang et al., 2014). On a second molecule in the asymmetric unit only amino acids gly101 through asp111 are evident in the electron density.

**Fig. 1:**
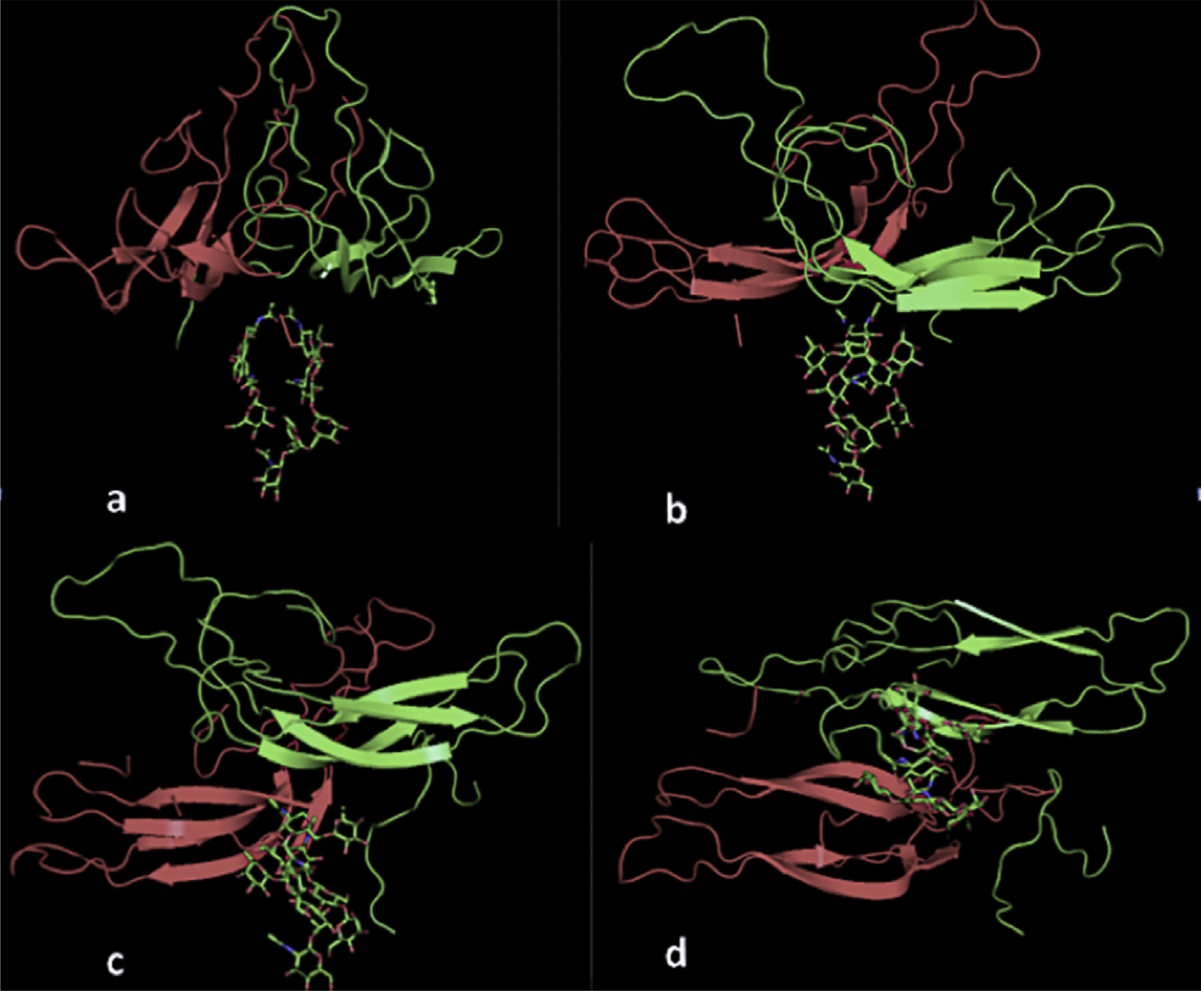
Four views of the asymmetric unit of the crystals, which are composed of NCS, two-fold related molecules of the β subunit of luteinizing hormone. In (a), (b) and (d) the NCS axis is evident, in (c) is an oblique view. The close association of the oligosaccharides, covalently attached at asn 13 (Baenziger and Green, 1988), from the two molecules is apparent.

It is unusual in most cases for oligosaccharides of a glycoprotein to be well ordered, but here that was not the case. On one molecule seven sugar residues were seen that conformed to the expected arrangement (Baenziger and Green, 1988), and on the other molecule five sugars (see supplementary Figure 2). Only the initial NAG bonded to an asn was present in the probe structure. In the asymmetric unit the oligosaccharides of the two molecules are closely apposed (Fig. 1), and even come into contact with one another, which may explain their stable conformations. The additional two sugars that are predicted, a mannose and a terminal sialic acid, are not present in the electron density, and are probably not disordered, but are most likely absent. Were they present, a clash with symmetry related molecules in the lattice would probably have resulted.

The oligosaccharides are on the opposite side of the β subunit from where the interface with the α subunit is expected. Hence the oligosaccharides would not interact with or contact the α subunit when bound. α And β subunits are similar in three-dimensional structure, both being “cysteine knots” with extended loops. The association of the β subunits in this asymmetric unit, however, is in no way similar to the organization of the α and β subunits in hCG or FSH.

The model of the asymmetric unit deposited in the PDB (entry 6P57) has bond length deviations of 0.009 ​Å, 1.80° bond angle deviations, and 0.084 ​Å^3^ deviations for chiral centers. There are no rotamer outliers (though 4 are in the low probability range) and there are 10 Ramachandran outliers (5 per molecule), with 80% in allowed regions, and 34 in generously allowed regions according to COOT. The number of Ramachandran outliers and marginal phi/psi angles appears elevated, but the molecule is unusual. As noted above, 10% of its residues are cysteines linked in disulfide bonds and 20% of the remaining residues are prolines. Thus, it is perhaps not surprising that unusual Ramachandran angles are frequent. Comparison of the geometry of the LH β model with the PDB models for hCG (1HCN) and FSH (1FL7) indicate that this LH β model is in fact an improvement in terms of geometry.

If the two NCS related β subunits in the asymmetric unit are superimposed upon the other (Fig. 2(a)), there are significant differences. While the cysteine knot core is virtually superimposable, the extended flexible loops show considerable variation. If polypeptide 5–100 of one β subunit is superimposed on the other β subunit the r.m.s.d. of main chain atoms is 1.47 ​Å. There are, in addition, a substantial number of side chain rotamers that differ between the β subunits within the asymmetric unit. If the two LH β subunit are each superimposed upon the β subunit of hCG, then the r.m.s.d. are 2.78 ​Å and 3.15 ​Å for main chain atoms using only the main chain atoms of peptide 5–100 for fitting. Fig. 2(b) shows the superposition of hCG β subunit (from 1HCN) on one of the two β subunits of LH β**.** Again, there is coincidence of the cysteine knot core, but substantial variation in the loop conformations.

**Fig. 2:**
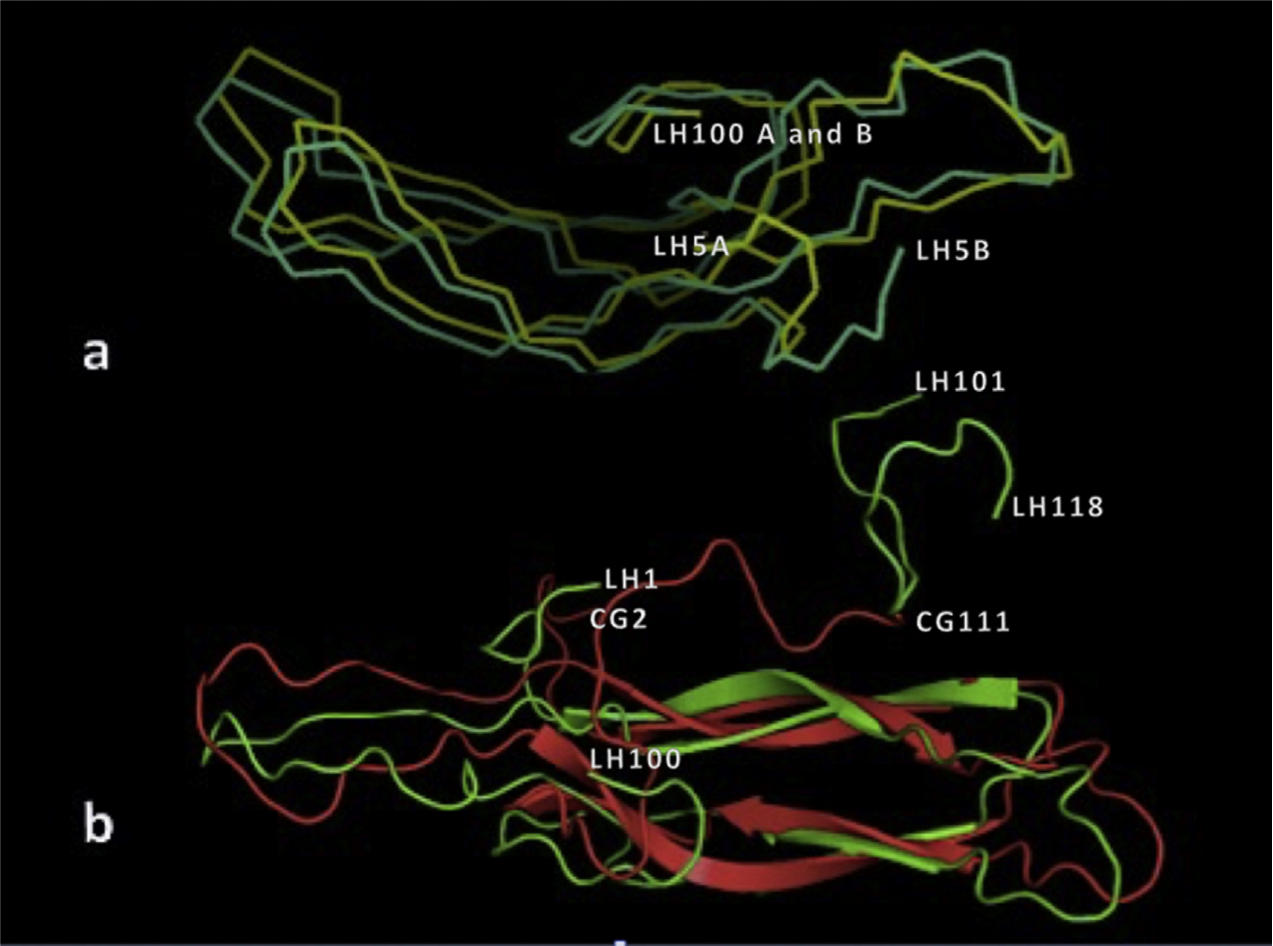
In (**a),** the C_α_ traces of two LH β subunits comprising the asymmetric unit of the crystals, in cyan and yellow, are superimposed showing the similarity of the two. Only main chain amino acids 5 through 100 were used for the superposition. The greatest differences between the two are at the amino and carboxy termini, and the segment 101 through 118, which was separated by proteolytic cleavage but remained linked to the body of the β subunit by the disulfide bond between cysteines 110 and 26. There are numerous differences in rotamer conformations between the two subunits as well. In (**b)** is shown the least squares superposition of a β subunit of luteinizing hormone in green upon the β subunit of human chorionic gonadotropin (from 1HCN). The similarity is strong in the cysteine – knot core, but it is evident that the extended, flexible loops diverge in conformation.

The α subunits of GPH are the same, and there is good evidence that the α subunit associates with the β subunit the same way in all four hormones (Jiang et al., 2014) (see supplemental Figure 3). Hence there is good reason to believe that the α subunit of LH probably interfaces with the β subunit of LH in the same way that the two subunits combine in hCG. If hCG is superimposed on an LH β subunit using only the hCG β subunit as guide, and the hCG β subunit then erased, the structure in Fig. 3 is produced. This should be a relatively accurate model for the intact LH.

**Fig. 3:**
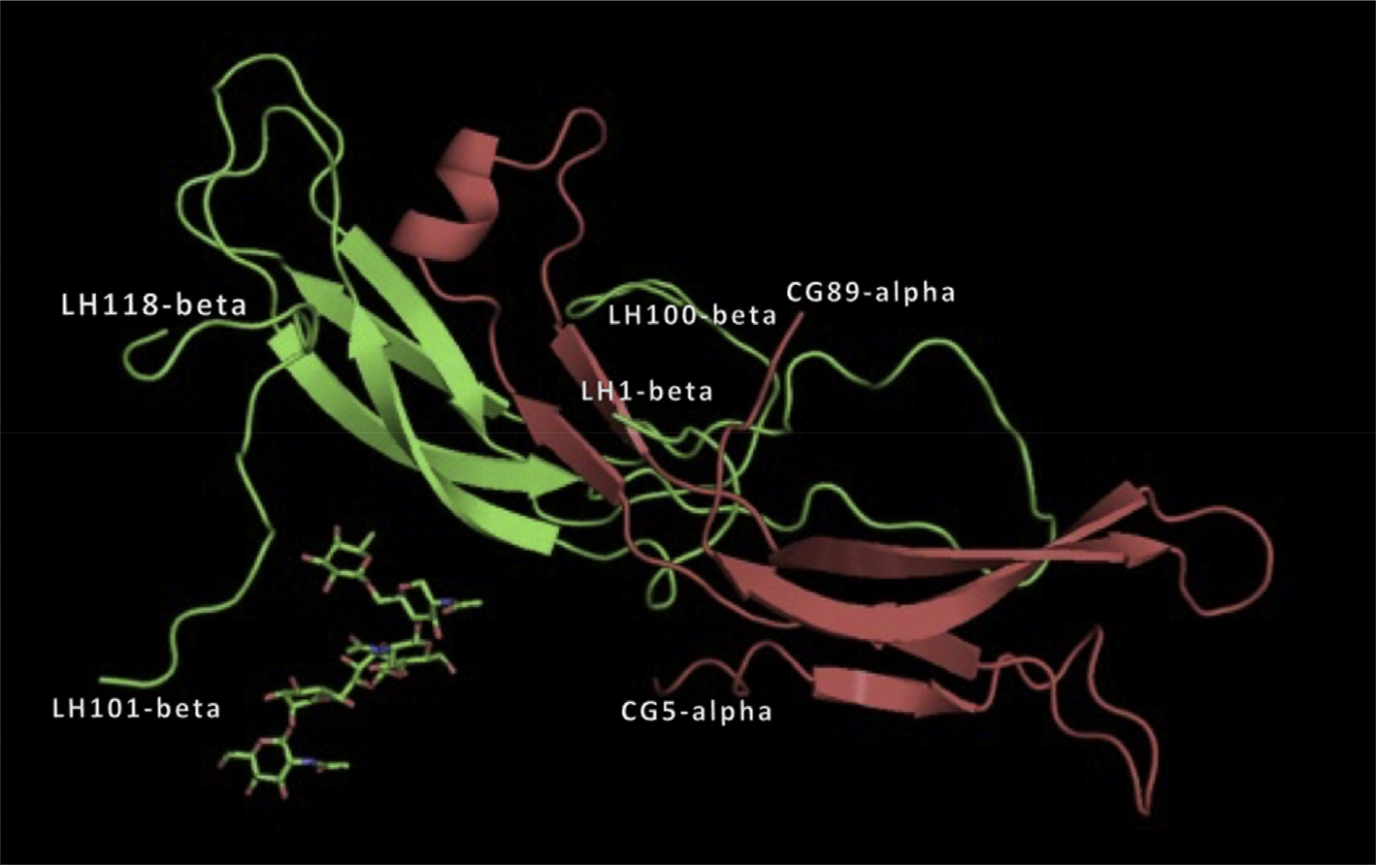
The α subunit in red, common to all of the GPHs, was placed by superimposing human chorionic gonadotropin (1HCN) upon a β subunit of luteinizing hormone using only β subunit amino acids 5 through 100, and the β subunit of hCG then erased. Thus, the image represents a model of the LH hormone, assuming the α and β subunit orientations are the same as in hCG, for which there is evidence (Jiang et al., 2014).

Superposition of hCG on the LH β subunit crystal structure, as described above, results in three clashes that might prevent association of the α and β subunits as in the model. These involve the amino terminal polypeptide of about 7 residues and the carboxy terminal fragment of the β subunit. These clashes could easily be relieved, however, by rearrangement of the relatively free, terminal strands upon dimerization. Thr98 of β overlaps val53 on α, but both are at the tips of flexible, extended loops, which also might reposition. The only other serious clash is tyr37 on β with ser34 on α**.** Relieving that clash would require assumption of a different side chain rotamer for tyr37 β and possibly a local rearrangement of the main chains of one, or both subunits.

A cautious effort was made to identify specific interactions in the LH model of Fig. 3 between the α and β subunits that might explain their association. The caution was, we felt, warranted chiefly for three reasons. First, the resolution of the β subunit model is only 3.16 ​Å, which alone makes assignment of interactions uncertain. Second, the model was constructed using a β subunit as it exists in the free state, and α subunit as it exists when paired with the β subunit of a different hormone, hCG. Finally, in the LH β subunit, the critical “seatbelt polypeptide” clearly has a non-native disposition. It is unlikely, therefore, that the orientations of side chains assigned to the model, at the interface, would be exactly the same as in the actual LH dimer. Indeed, NMR studies of the free α and β subunits, and the intact heterodimers indicate that the free subunits are less ordered, and exhibit more fluid conformations. This further suggests that heterodimer formation includes at least some local conformational rearrangements (Jiang, et al., 2014).

Inspection of the α/β interface of the model in Fig. 3 reveals, however, a number of possible or potential interactions, most involving hydrophobic contacts between amino acid side chains. In particular, these include val44 β and pro46 β with phe17 α and phe18 α, a cluster that forms a small, internal hydrophobic core in the heterodimer. Also present are hydrophobic interactions between ile33 β with both tyr37 α and pro38 α. The disulfide bridges of cys32 α - cys 84 α, and cys10 α - cys60 α are closely apposed to leu5 β and form another such cluster.

Hydrogen bonds would be risky to predict at this resolution, but two prominent salt bridges are apparent. These are between the side chains of lys42 β and glu21 β with glu27 α and lys45α respectively. The only apparent common feature shared by LH and hCG heterodimer interactions is the hydrophobic cluster in hCG involving leu45 β and val76 β with tyr18 α and phe74 α that is noted above for LH, that is, val44 β and pro46 β with phe17 α and phe18 α.

## Conflicts of interest

None declared.

## Figures and Tables

**FigS 1:**
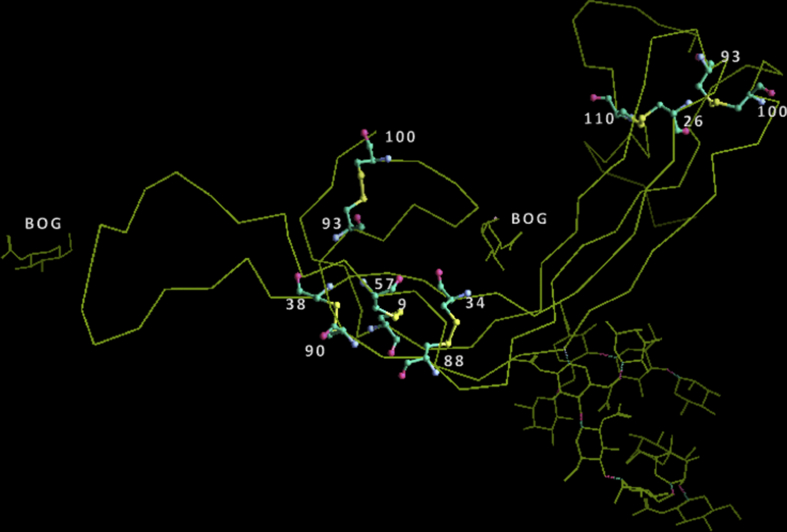
In yellow is a tracing of a β subunit of luteinizing hormone with its linked oligosaccharide. Shown in ball-and-stick representation are the six disulfide bridges that produce the cysteine – knot structure. The numbers represent the cysteine sequence numbers. The disulfide bridges are 9-57, 23-72, 26-110, 34-88, 38-90, 93-100.

**FigS 2:**
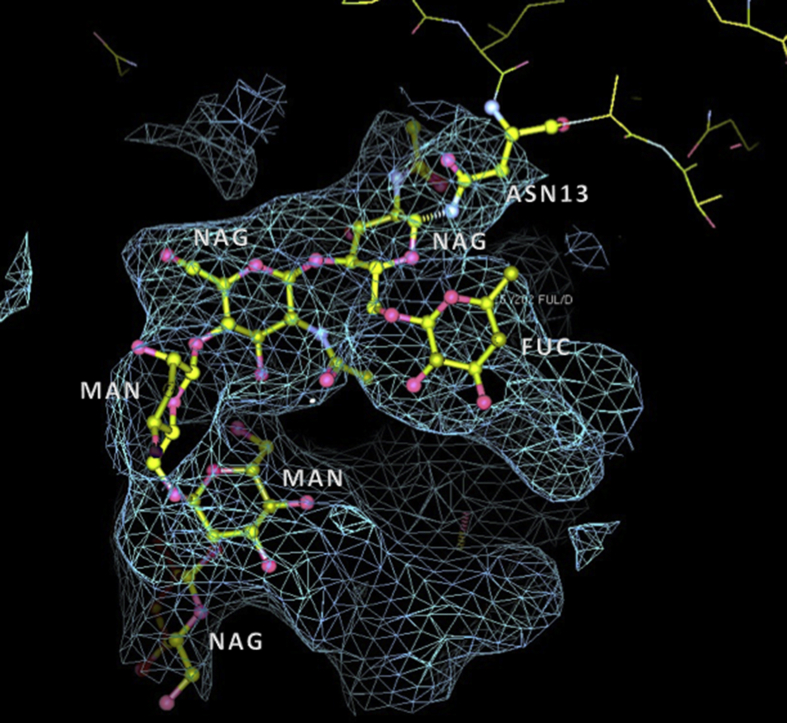
The seven sugar residues attached by N linkage to asn 13 of one beta subunit is shown superimposed on their electron density. The electron density in the lower right that contains no model is filled by sugar residues of the oligosaccharide of the second β subunit (not shown) in the asymmetric unit.

**Figs 3:**
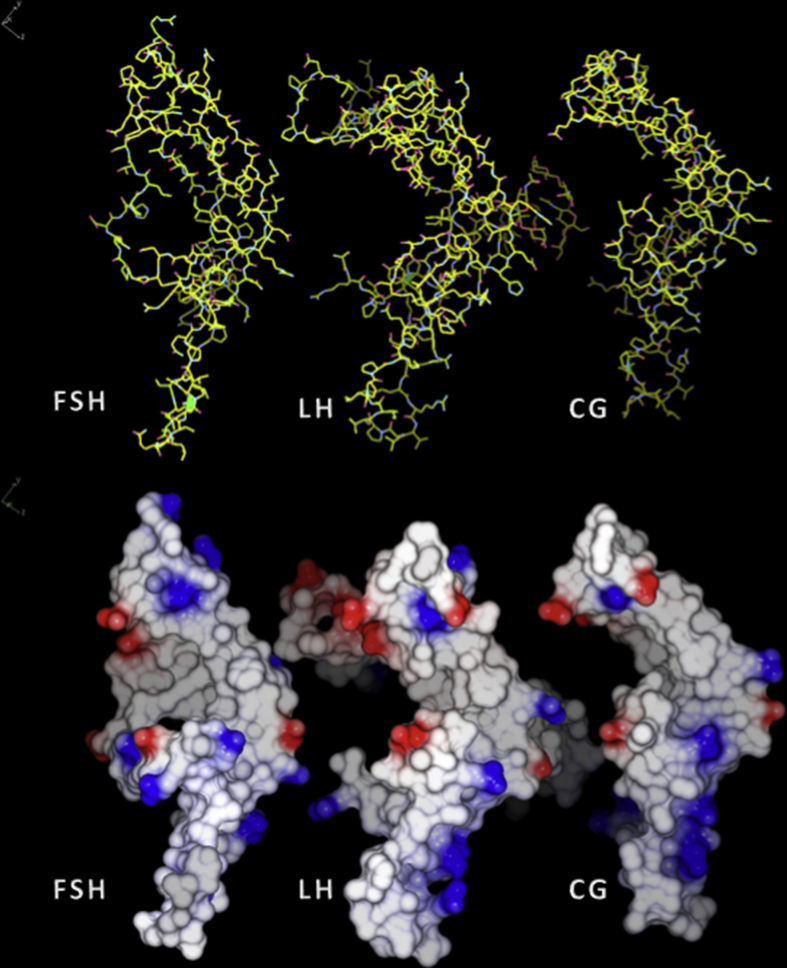
At top are all atom representations, from left to right, of the β subunits of follicle stimulating hormone (FSH), the luteinizing hormone model (LH), and human chorionic gonadotropin hormone (CG). In (b), in the same order, the hormone subunits are presented with electrostatic surfaces. The hormone β subunits of FSH and CG have the “seatbelt polypeptide” as found in the native structure, while in LH, the proteolytically cleaved “seatbelt polypeptide”, seen in the upper left, is held to the core of the subunit only by a disulfide bond.
